# Possibility of High-Speed Ultrasonic Detection of the Internal Material Defects in Rails

**DOI:** 10.3390/ma19010028

**Published:** 2025-12-20

**Authors:** Leszek Chałko, Łukasz Antolik, Mirosław Rucki, Miroslav Trochta

**Affiliations:** 1 Faculty of Mechanical Engineering, Casimir Pulaski Radom University, Stasieckiego 54, 26-600 Radom, Poland; leszek.chalko@urad.edu.pl; 2 Railway Research Institute, Chlopickiego 50, 04-275 Warsaw, Poland; lantolik@ikolej.pl; 3 Institute of Mechanical Science, Faculty of Mechanics, Vilnius Gediminas Technical University, 11 Sauletekio al., LT-10223 Vilnius, Lithuania; 4 Faculty of Mechanical Engineering, VSB-Technical University of Ostrava, 17. listopadu 2172/15, 70800 Ostrava, Czech Republic; miroslav.trochta@vsb.cz

**Keywords:** non-destructive testing, rail monitoring, ultrasonic inspection

## Abstract

**Highlights:**

**What are the main findings?**

- At higher speeds, the quality of acoustic coupling in ultrasonic railway inspection systems is highly dependent on the applied fluid flow;
- The pressing force exhibited a less significant impact on the acoustic coupling at high speeds.

**What are the implications of the main findings?**

- Proper control of the liquid flow rate and the pressing force makes it possible to keep reliable ultrasonic measurements at high speeds;
- The experimental results indicated that it was possible to keep the high quality of the ultrasonic signal at speeds of 120 km/h, which is three times higher than usual;
- Flaw detection of working rails is possible in short time spans between fast passenger trains.

**Abstract:**

Quick and reliable in situ non-destructive assessment of the material structure is especially critical in the case of measurement of rail defects concerning the demands of quick, uninterrupted transportation and safety. This paper presents the test results of a patented measuring head that is able to perform ultrasonic rail defect detection at speeds of up to 120 km/h. The experimental data was collected and discussed. Statistical analysis was performed in terms of bottom echo drop as a function of velocity, pressing force, and film thickness between the sensor and the rail material surface, as well as the coupling fluid stream intensity. The results proved the feasibility of the device for usage at high speeds for the state monitoring of rails in service.

## 1. Introduction

The railway communication and transportation system plays a critical role in the modern world. An industrial railway track can be loaded with and transport ca. 8,000,000 tons over 5 years of exploitation [1]. Sustainable, safe, and reliable railway operation is possible only when efficient maintenance of the rails in service is performed in a timely manner [2]. In particular, the track geometry and degradation level of the respective materials should be carefully monitored and controlled [3,4], and supervision, optimization, and tuning can be performed with the application of Artificial Intelligence (AI) algorithms [5,6]. There are many studies focused on train sequencing and routing, as well as the timing decisions concerning short-term maintenance in a working railway network [7].

A wide range of conventional nondestructive testing methods are available, including X-ray and ultrasonic techniques [8]. Ultrasound has been used for the real-time monitoring of the rails and demonstrated good sensitivity to cracks across the material of the rail head, web, and foot [9]. Appiani and Carboni demonstrated that higher-frequency electromagnetic acoustic transducers outperformed the lower-frequency electromagnetic acoustic transducers in the ultrasonic rail inspections, exhibiting higher flaw detection rates [10]. The effectiveness of the ultrasonic equipment on axle cracks can be expressed as probability of detection (POD). It is influenced by numerous factors, including the crack geometry, size, location, and orientation; the geometry and composition of the axle structure; the techniques and methods employed for the detection; and the level of automation of the equipment used for the measurement [11]. Moreover, it was demonstrated that the rail surface roughness and the applied coupling media influence the results of ultrasonic flaw tests considerably [12]. Due to the variations in defect lengths between zones, real-time combined data on ballast and moisture was proposed to be integrated with ultrasonic sensing, with the application of AI-based management [13].

Sliding holders with two or three ultrasonic probes are typically used for ultrasonic non-destructive inspection [14]. Mackiewicz et al. [15] proposed a reliable model and program for the precise calculation of losses for both normal beam probes with longitudinal waves and for commonly used angle beam transducers that emit transversal waves. Ranachowski with co-authors attempted to optimize the operational conditions of the ultrasonic detector, ensuring the laminar flow of the coupling liquid, and to take advantage of the aquaplaning effect [16].

It should be noted that the ultrasonic method is a volumetric method, unlike the surface methods based on visual inspection [17], laser imaging [18], laser interferometry [19], or differential topographic images [20]. Ultrasonic inspection makes it possible to reveal developing flaws preventively, while visual methods can detect only existing cracks and damages. It can be stated that surface inspection demonstrates how the rail is worn if flaws have not been detected preventively.

The main challenge of ultrasonic measurement of rail material internal flaws is related to high-speed movement. In order to identify flaws automatically in the rail foot, [21] an innovative ultrasonic system has been designed, with spray water technology as a coupling method ensuring optimal transmission of signals and their subsequent reception. The tests were performed with a running speed of approximately 1 m/s, corresponding to the average human walking speed. According to available data [22], visual camera-based methods can reach an inspection speed of 320 km/h, while conventional ultrasonic devices can operate only at speeds from 40 to 80 km/h, with typical real speeds of 15 km/h, since it is challenging to detect deep flaws over 4 mm using high-speed ultrasonic systems.

In the present study, a typical probe block with a slight modification was used for determining acoustic signal losses during measurement at high speeds. In an inspection of ultrasonic material defects, a mechanical wave signal is generated with a piezoelectric transducer. After the wave enters the tested material, the echo reflected from a flaw located deep within the material is received and analyzed. In order to transmit the waves from a probe to a sample, it is necessary to fill the slot between the transducer and the material’s surface using various coupling agents [23,24]. The coupling liquid minimizes energy losses of the ultrasonic wave during its passage between the transducer and the material being tested [25]. During static measurements, the coupling area between the probe block and rail surface filled by the fluid is stationary in its cross-section. However, the thickness of the coupling layer cannot be controlled precisely, and thus even stationary thickness generates signal energy losses and subsequent measurement errors [26]. This is much more apparent in the case of relative motion between the probe block and the tested rail surface, where distance and respective layer thickness change dynamically and turbulences and gaseous bubbles appear in the coupling fluid, increasing noise and measurement errors. Specifically, the recently published paper [27] describes a refined imaging technique based on wavenumber-domain plane waves that enables dynamic imaging in real time. The authors made simulations and experiments with vertical vibrations of the probe, typical for the rail measurement, but did not address signal losses in the coupling layer. Since dynamics of the coupling film thickness may result in serious errors, the present work is dedicated to signal losses during dynamic flaw detection in rails.

The results presented below demonstrate that under certain conditions, the ultrasonic internal flaw measurement of rails is possible at high speeds of up to 120 km/h. The experiments included innovative measurement of distance, as well as control of the coupling fluid rate and pressing force as the main factors affecting acoustic signal losses.

## 2. Materials and Methods

### 2.1. Ultrasonic Detector

**Figure 1:**
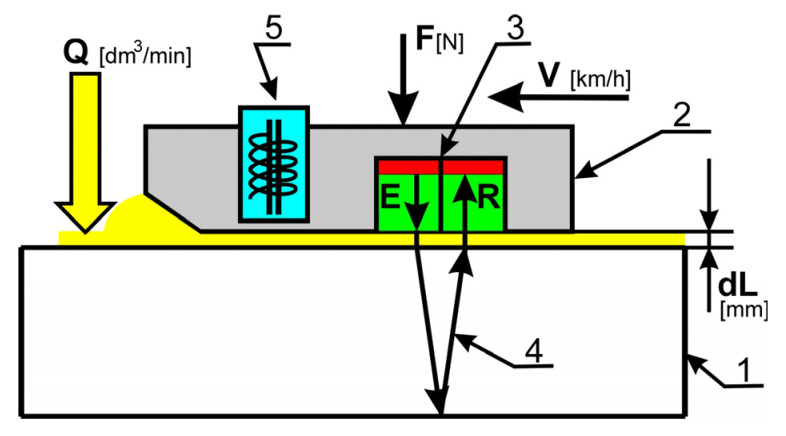
Scheme of the device for the internal flaw detection in rails. 1—Tested rail; 2—Block containing the ultrasonic probes; 3—Double ultrasonic probe 2.5 MHz; 4—Path of the ultrasonic waves; 5—Inductive proximity sensor.

A scheme of the flaw detection principle with the proposed ultrasonic device is shown in Figure 1. It consists of a piezoelectric ultrasonic transducer with emitter and receiver denoted E and R, respectively. The movement direction with velocity *V* [km/h] and pressing force *F* [N] are shown with respective arrows. Cold water applied as coupling fluid is supplied at a flow rate *Q* [dm^3^/min], forming a film of thickness dL between the block (2) and rail surface (1). The innovative component of the system is the proximity sensor (5) used for continuous measurement of the distance between the surfaces and the respective coupling layer thickness *dL*.

**Figure 2:**
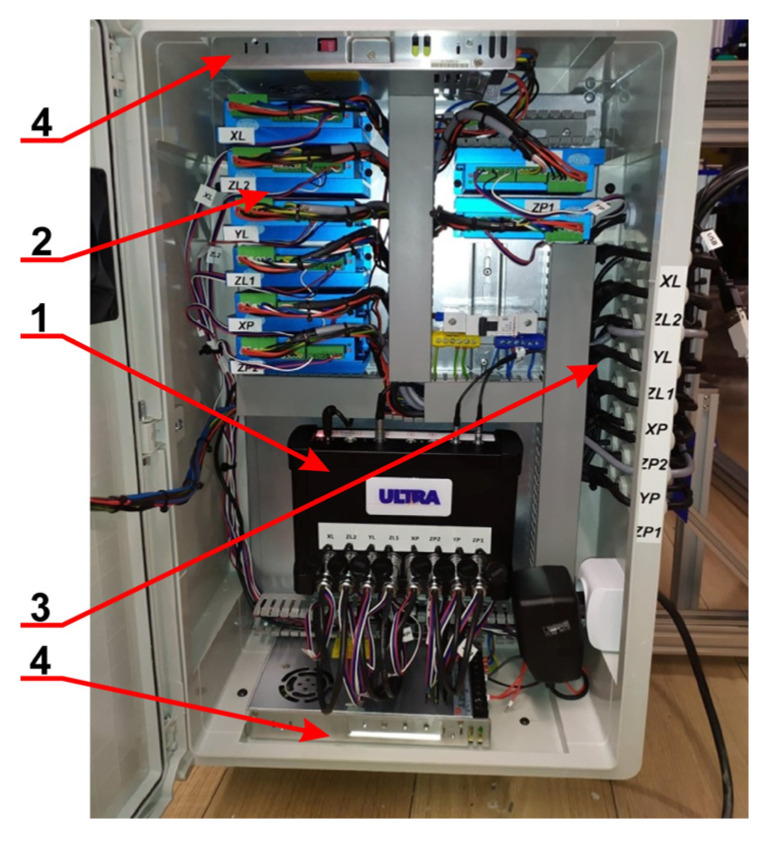
Flaw detector used in the experiments. 1—HUB; 2—FPGA module; 3—Signal inputs; 4—Power supplies.

The flaw detector and ultrasonic transducers were delivered by ZBM ULTRA Sp. z o.o. (Nadolice Male, Poland). The detector shown in Figure 2 was designed for high-speed internal flaw detection in rails, and exhibited the following characteristics:

- Pulse repetition frequency (PRF) 5 kHz;
- Refresh frequency DAC (TCG) > 250 kHz;
- Amplitude of emitter pulse voltage 400 V;
- Middle range frequency *f*_0_ between 1.5 and 3 MHz;
- Broadband Δ*f* up to 2 MHz.

The flaw detector made it possible to record the oscillogram A-Scan continuously, which was used as a display mode.

### 2.2. The Test Rig

Measurement of flaws with a velocity of 120 km/h would require a very long linear rail with known defects, which is inconvenient for laboratory tests. Thus, for the current research, the test rig designed for friction pairs (brakes) available at the Railway Research Institute (Warsaw, Poland) was adopted. In order to imitate a rail, a specially prepared railway wheel with a proper contact surface was used.

**Figure 3:**
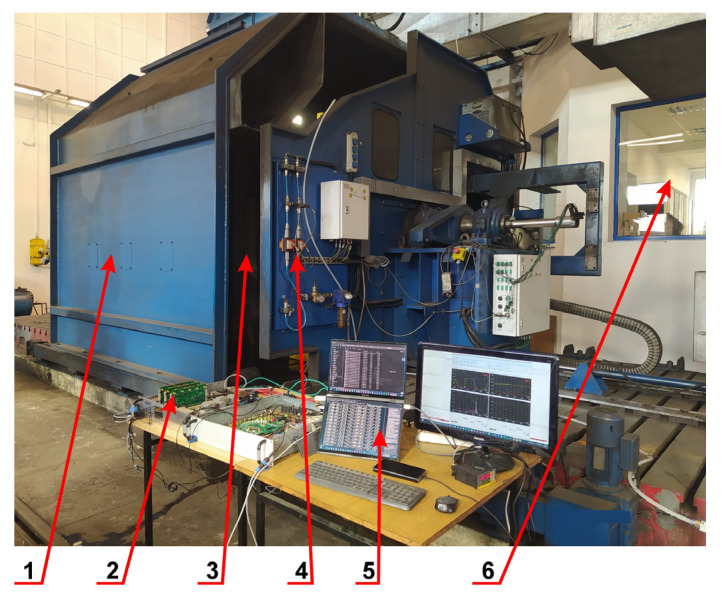
The test rig adopted for the high-speed flaw detection testing. 1—Drive system, 2—Measurement apparatus with a multichannel ultrasonic flaw detector; 3—Measuring chamber with the test wheel; 4—System for supply and measurement of the coupling fluid; 5—Computerized control and recording unit of the ultrasonic apparatus; 6—Control unit for the driving system of the test rig.

The test rig design is shown in Figure 3. It allowed for measurements with the following parameters:

- Maximal linear velocity was 420 km/h for the wheel surface points, wheel diameter was Ø890 mm;
- Maximal rotational speed of the shaft was 2500 rpm;
- Driving motor power was 536 kW at 1150 rpm;
- Torque at speeds below 1150 rpm was 4450 Nm.

The digital control system used in the test rig enabled smooth adjustment of the spindle rotation speed, maintaining constant speed under the load. The entire system had measurement traceability for key parameters and UIC (International Union of Railways) homologation.

For the measurement of the thickness of the coupling film denoted by *dL* in Figure 1, an inductive proximity sensor was used. It was delivered by Balluff (Neuhausen, Germany), BAW0067 type, measurement range 0.2–7 mm. The sensor was calibrated according to the manufacturer’s instructions. A digital dynamometer was used to measure tension force of the compression springs.

To measure the flow rate of the coupling fluid supplied during the test, a manually adjustable flow control valve was used. A liquid flow meter type PM-1/4-B (Termipol, Lubliniec, Poland) was equipped with a pulse counter Termipol Cl8 integrated with a digital recorder. The device enabled the measurement of flow rates as low as 0.25 L/min, providing the measurement signal of 7055 imp/L ± 10%. The number of pulses was proportional to the flow rate precisely controlled in the laboratory conditions. It was proved experimentally that in the real conditions of movement along the railway, the system was able to control the flow rate with an absolutely satisfactory accuracy of ± 0.1 dm^3^/min.

In the experiment, a computer-based measurement system with a data acquisition card USB-4716 by Advantech (Taipei, Taiwan) was used to record the data. It was able to operate with a frequency of up to 200 kHz, registering synchronous values of flow rate *Q* and coupling agent thickness *dL*. For each dataset, linear velocity *V* and pressing force *F* values were constant. Resulting values of the returning echo amplitudes of the emitted ultrasonic wave were recorded in the operating system of the flaw detector.

In order to adapt the test rig to high-speed flaw detection experiments, it was necessary to design and assemble the following system components:

- An imitation of a long rail in the form of a test wheel;
- A frame allowing the probe block to be guided precisely along the surface of the test wheel;
- A system for control, supply, and measurement of coupling fluid;
- A digital measurement chain.

**Figure 4:**
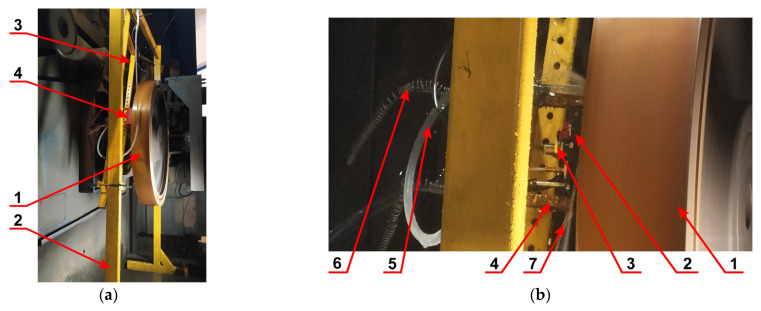
Details of the adopted test rig: (**a**) Test wheel mounted in the frame; (**b**) Position of the probe block and fluid supply. Explanations are given in the text.

The frame was designed in a universal way, so that it could be assembled and guide the probe block along the test wheel, as shown in Figure 4a. Here, the numbers denote the following components: 1—test wheel of diameter Ø950 mm; 2—main frame; 3—auxiliary frame; 4—probe block. The details of the measurement system are shown in Figure 4b, as follows: 1—test wheel; 2—probe block; 3—inductive proximity sensor for dL measurement; 4—guide for the probe block; 5—coupling fluid supply; 6—tension springs for pressing the probe block with adjustable force; 7—splashes of coupling fluid with cavitation effect.

It should be noted, however, that the area between the probe block and the wheel did not reflect the dynamics of real movement when changing the shape and thickness of the coupling layer. The natural changes in the rail running surface shape and the continually worn probe block surface, as well as rail waviness, screw joints, railway turnouts, etc., will undoubtedly affect measurement results. However, for the present research, it was necessary to make simplifications, which require further consideration after the successful completion of the current stage of investigations.

### 2.3. The Test Parameters

During the test, the alterations in the signal value *dKd* were registered. These alterations represented a backwall echo reflected from the test wheel dependent on coupling fluid thickness dL, linear velocity *V*, pressing force *F*, and fluid flow rate *Q*, i.e., *dKd*(*dL*, *V*, *F*, *Q*). The respective values were set as follows:

- Linear velocity *V* = 0 ÷ 135 km/h;
- Pressing force *F* = 12.8 ÷ 46.1 N;
- Coupling fluid flow rate *Q* = 0 ÷ 2.4 dm^3^/min.

The distance dL between the transducer and the tested material representing the thickness of the coupling liquid layer and the value *dKd* [dB] of the ultrasonic signal reflected back to the transducer from the opposite surface was registered during the test. The results of the ultrasonic signal measurement and the distance value for the static state, i.e., at a speed of 0 km/h, were used as reference values.

## 3. Results and Discussion

**Figure 5:**
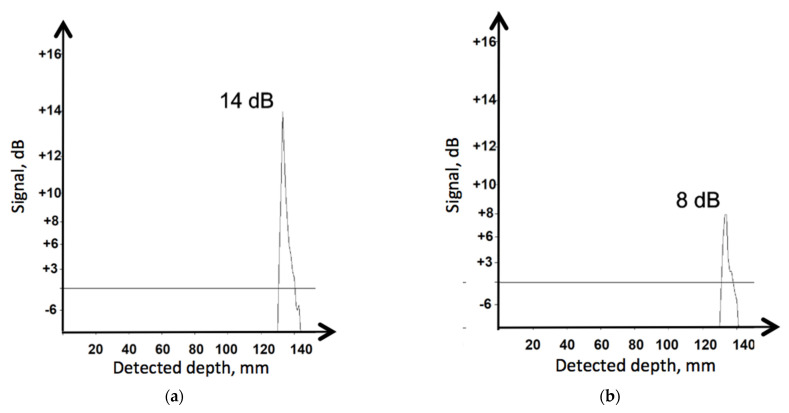
Reduction of the recorded signal is dependent on speed: (**a**) *V* = 0 km/h; (**b**) *V* = 120 km/h.

In order to visualize the disturbance effect of high-speed movement, the detected A-Scans are presented in Figure 5. They illustrate the two extreme situations, when the probe block is not moving, *V* = 0 km/h, and when the speed is maximal *V* = 120 km/h. All other parameters remained the same, as follows: pressing force *F* = 30 N and coupling fluid flow rate *Q* = 0.3 dm^3^/min. The peak corresponds to the echo depth of 134 mm. A visible reduction in the recorded signal value is observed, caused by the increase in the transducer’s relative speed along the tested material. In fact, a reduction by 6 dB in this case can be considered a very good result. No other significant deformations in the recorded signal diagrams were noted. Application of range gain control made it possible to eliminate the noise and limit signal analysis only to the tested distance range relevant for the present research.

The registered measurement results were processed with *Statistica* software, using the ANOVA method with a regression model that considered both linear and non-linear components. Table 1 contains the results of approximation of *dKd* with the function:

y = a + bx + cx^2^ (1)

where a, b, and c correspond to the respective coefficients for functions describing *dKd*(*V*), *dKd*(*F*), *dKd*(*Q*), and *dKd*(*dL*). The results were given within a 95% confidence interval; coefficient of determination was R^2^ = 0.8529, Standard Error of Estimate was 0.82544, and Residual Mean Square was 2.213616.

The complete regression equation describing the dependence of acoustic signal loss values *dKd* on the measured conditions of the coupling fluid film formation is presented below:

*dKd* = −4.056 − 1.090*V* − 4.423*V*^2^ + 0.711*F* + 1.505*F*^2^ − 7.179*Q* − 3.847*Q*^2^ − 2.611*dL* −2.775*dL*^2^ + 1.103*VF* + 2.709*VQ* + 10.324*VdL* + 5.018 FQ − 0.226*FdL* − 7.610*QdL*. (2)

Statistically significant components are emphasized in bold. After all insignificant components are removed, the equation can be written as follows:

*dKd* = −4.056 − 4.423*V*^2^ − 7.179*Q* − 3.847*Q*^2^ + 10.324*VdL* + 5.018*FQ* − 7.610*QdL*. (3)

Linear velocity above 33 m/s or 120 km/h between the probe block and the measured surface is a significant parameter, since the dynamics of measurement should be kept in continuous contact with the rail and simultaneous durability of the probes. Otherwise, the measurement is not reliable and maintenance expenses are too high. The dynamic component is necessary to ensure required contact despite alterations in the rail’s profile, and to omit obstacles without damage to the probe block. Thus, it was necessary to reduce the mass of the block and to avoid shock absorbers because the latter would set limitations on the quick jump in the event of a collision with a damaged rail joint or when passing a railroad switch. The increased pressing force fulfilled the requirement of continuous contact with the measured surface in the upper area of the measuring range, ensuring a quick return to normal work conditions after a momentary disruption as mentioned above.

However, abrasive wear of the probe block sets the opposite limitation for the pressing force, shortening its service time significantly. To prevent the block from wear and tear, wear-resistant material can be applied. However, it was found to be more favorable to balance between relatively cheap material and regular replacement of worn probe blocks.

**Figure 6:**
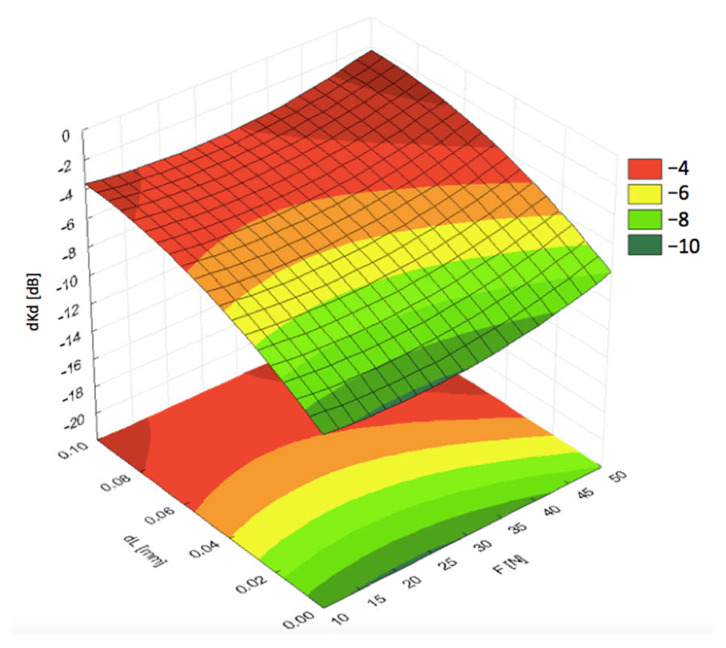
Dependence of the signal decrease (*dKd*) on the distance (*dL*) and pressing force (*F*) at linear speed *V* = 120 km/h.

**Figure 7:**
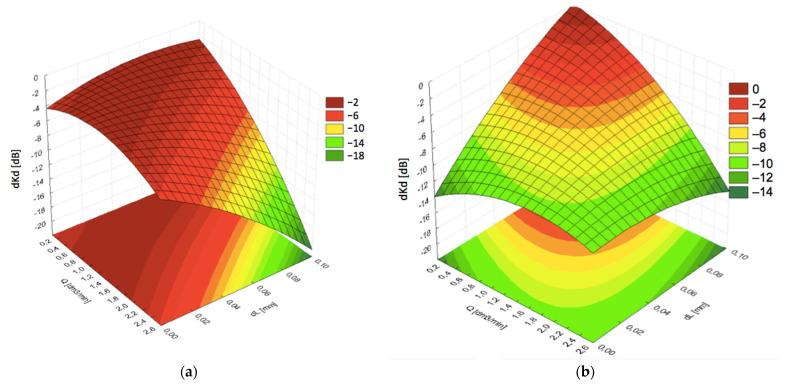
Dependence of the signal decrease (*dKd*) on the distance (*dL*) and coupling fluid flow rate (*Q*) at different linear speeds: (**a**) *V* = 40 km/h; (**b**) *V* = 120 km/h.

**Figure 8:**
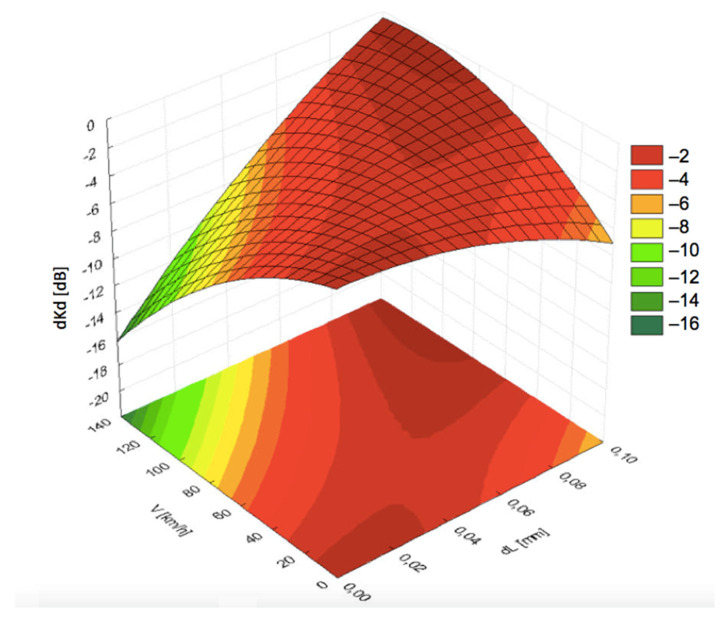
Dependence of the signal decrease (*dKd*) on the distance (*dL*) and linear speed (*V*) under pressing force *F* = 30 N and coupling fluid flow rate *Q* = 0.3 dm^3^/min.

**Figure 9:**
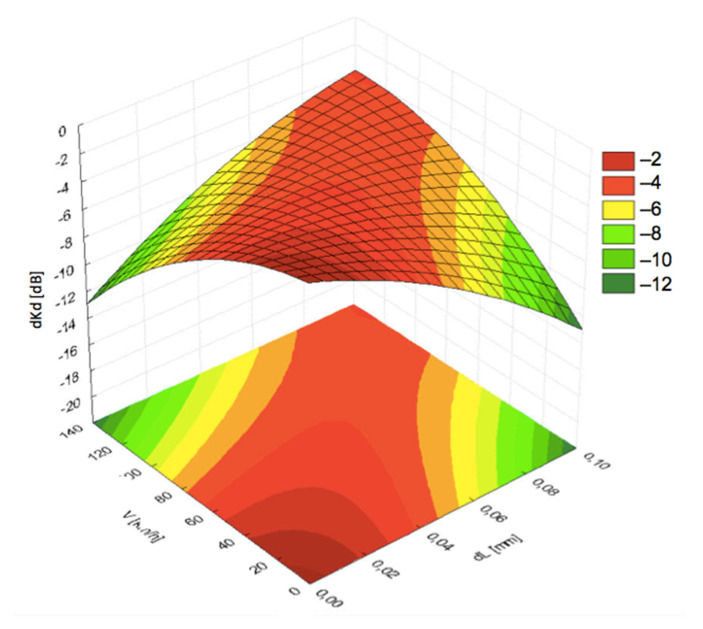
Dependence of the signal decrease (*dKd*) on the distance (*dL*) and linear speed (*V*) under pressing force *F* = 30 N and coupling fluid flow rate *Q* = 1.25 dm^3^/min.

**Figure 10:**
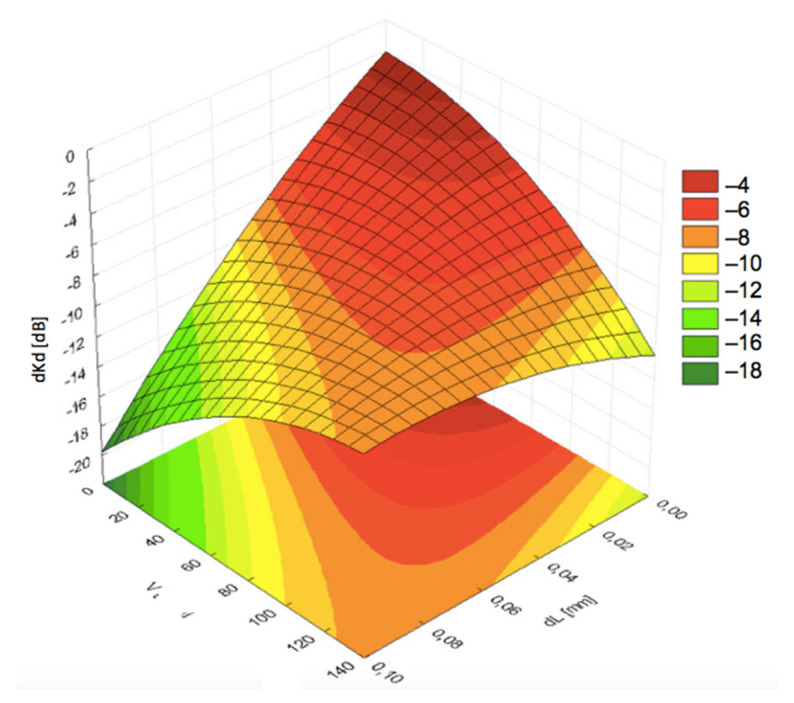
Dependence of the signal decrease (*dKd*) on the distance (*dL*) and linear speed (*V*) under pressing force *F* = 30 N and coupling fluid flow rate *Q* = 2.0 dm^3^/min.

Visualization of the results with 3D diagrams is presented in Figure 6, Figure 7, Figure 8, Figure 9 and Figure 10. It can be seen from Figure 6 that the pressing force *F* exhibited less significant impact on the acoustic coupling at high speeds, compared to the impact of fluid film thickness *dL*. Thus, the pressing force can be treated as a priority in the dynamic analysis of measurement conditions.

The experimental results indicated that pressing force between 15 N and 45 N had a very small impact on signal losses in the coupling layer. In fact, the pressing force itself was dependent on the two contradictory conditions of interaction between the probe block and the tested rail. On one hand, the force should be increased to keep optimal dynamics of the probe block and the rail, especially when it comes to returning to the measurement position after encountering an obstacle. On the other hand, the force should be decreased in order to avoid excessive wear of the probe surface. From the experimental data it was assumed that the second condition prevailed and the optimal pressing force should be ca. 15–20 N. The planned tests in real railway conditions are supposed to verify this assumption.

Another important parameter was the consumption of the coupling liquid. The limitations are related to the capacity of water tanks and availability of water along the road. With the end of the steam locomotive era, the number of water supply points available for rail vehicles decreased significantly. On the other hand, it was found that the amount of water had a considerable effect on the signal losses. Graphs presented in Figure 7a indicated that the optimal water consumption depended on the film thickness dL at lower speeds of the examined probe block. The optimal flow rates were found to be in the middle area of the measurement range, i.e., ca. 1.5 dm^3^/min, which can be considered a low consumption level.

However, an increase in the linear speed required limiting the amount of water supplied to a minimum in order to keep acoustic signal losses low, as shown in Figure 7b. Simultaneously, continuous contact and sufficient distance *dL* had to be kept. As a result, a smaller pressing force *F* is required between the probe block and the rail, to leave a thicker coupling fluid layer *dL*.

In typical measurement systems working at smaller velocities, losses of the signal caused by worsened propagation of acoustic waves through the coupling layer depend on the flow rate only to a small degree. Flow rates between 0.4 and 2.0 dm^3^/min ensured the optimal coupling conditions. The function expressed by Equation (3) suggests that at higher velocities the effect could be different. Figure 8, Figure 9 and Figure 10 present the graphs for signal decrease *dKd* dependent on the flow rate *Q*. For comparative study, the results are shown for the same pressing force 30 N and respective flow rates of 0.3 dm^3^/min, 1.25 dm^3^/min, and 2.0 dm^3^/min. The results suggested an unfavorable effect of flow rate increase. Figure 9 indicated relatively low losses in *dKd* for all values of velocity *V* from 0 up to 135 km/h under the condition of larger thickness *dL*. The graphs in Figure 9 and Figure 10, corresponding with larger flow rates, exhibited a significant decrease in the signal level with increasing linear velocity *V*.

Presumably, an increase in the velocity promoted the dynamic processes of coupling layer formation due to the viscosity of the liquid. That would explain the layer thickness formation of 0.08–0.09 mm at small flow rates 0.3–0.4 dm^3^/min. Similar values of *Q* are appropriate for lower velocities too, when the thickness *dL* is smaller. The desirable effect can be strengthened through the respective shaping of the area between the rail and the probe block, which is the subject of future research aimed at minimizing splashes of the liquid.

Moreover, it was observed that at higher speeds, especially at 120 km/h, a significant amount of the coupling liquid was splashed by the air streams. Forceful air streams generated by the movement caused losses of the coupling liquid, disturbing the mutual dependence between the flow rate and fluid layer thickness. Upcoming research is dedicated to the aerodynamic conditions of coupling layer formation and minimization of the losses.

Notably, the coupling fluid used in this study was water, which appeared to be suitable from ecological and economical perspectives. Some addition of surfactants such as dishwashing liquid helped to reduce surface tension and thus improved the wetting of the rail surface. As a result, the effect of the coupling fluid on traffic safety, in particular the extension of the braking distance of vehicles, is comparable to the effect of a light rainfall.

When interpreting the ultrasonic wave beam, it is usually considered to be rectilinear, while in reality it is divergent. Taking into account the shape of the beam and the random variability of the inclination angle of the reflecting flaw surface, it can be seen that the sampling frequency of 6 kHz ensured the test repetition ca. every 5.5 mm at 120 km/h. This allows for statistically reliable detection regardless of the beam angle within the 0–70 degree range, which has been demonstrated elsewhere [15]. The value of the echo signal drop in the coupling layer is not significantly dependent on the testing angle. It depends mainly on the condition of the coupling layer, including its continuity and homogeneity.

The results demonstrated that the proposed system allowed for non-destructive ultrasonic measurement of rail flaws at high speeds. The decrease in the ultrasonic measurement signal was kept at an acceptable level, enabling reliable flaw detection at a linear speed of at least 120 km/h. Proper control of the distance *dL* was possible with the right pressing force *F* and coupling fluid flow rate *Q*.

The methods used so far in the inspection of railway tracks with guided ultrasonic probe blocks and coupling fluid have proven effective at low speeds, not exceeding 60 km/h. However, with increasing speed, the flow in the contact zone is disrupted, making it challenging to control the parameters affecting the interaction between the transducer block and the rail, and thus to minimize measurement signal losses. The experimental results indicated that it was possible to maintain a high quality of the ultrasonic signal at speeds two or even three times higher.

The authors are aware that the presented research results are subject to certain errors due to the simplified conditions of interaction between the working surface of the ultrasonic transducer and the surface being tested. Moreover, the effects of fluid viscosity may be altered in real-world conditions compared to the laboratory ones. These errors need to be identified and corrected when transferring the system to real-world conditions of measurements on railway tracks.

Nevertheless, the obtained results allowed for a significant simplification of the measurement system. For example, it is possible to eliminate the remote control of the variable contact force of the transducer blocks against the rail. Implementing a variable coupling fluid flow rate dependent on the measurement speed allows us to obtain correct inspection results.

## 4. Conclusions

The ultrasonic flaw detection in working rails at high speeds (above the standard 60 km/h) requires proper control of acoustic coupling between the transducer and the examined material. Based on the measurement results, a complete regression equation of the dependence of acoustic signal loss on the conditions of the coupling fluid film formation was determined. It was found that the signal quality was highly dependent on the applied fluid flow *Q* that filled the slot between the probe block and the rail. An increase in flow rates above 0.3–0.4 dm^3^/min caused extensive losses of the acoustic signal. In typical measurement systems working at lower velocities, losses of the signal caused by worsened propagation of acoustic waves through the coupling layer depend on the flow rate only to a small degree. At the same time, the effect of the pressing force in the examined range between 15 N and 45 N was found to be very small. The experimental results suggested an optimal force range of ca. 15–20 N.

The obtained results demonstrated a possibility of keeping low signal energy losses at high speeds through proper control of the coupling fluid flow rate. Further research and implementation of the proposed solution will enable flaw detection at higher speeds up to 120 km/h, which is three times higher than the one typically used at present. Thus, flaw detection in working rails will be possible in short time spans between fast passenger trains, significantly improving maintenance procedures and railway transportation safety.

## Figures and Tables

**Table 1:** Results of the statistical analysis.

Variable	Coefficients in Equation (1)	Value	Std. Dev.	*t*-test t(75)	Significance *p*	Conf. −95%	Conf. +95%	Regression Coefficient	Std. Dev.	Conf. −95%	Conf. +95%
all	a *	−4.056	0.684	−5.934	0.000	−5.418	−2.694	−4.056	0.684	−5.418	−2.694
*V*	b	−1.090	1.311	−0.831	0.408	−3.701	1.522	−0.545	0.655	−1.851	0.761
	c *	−4.423	1.053	−4.199	0.000	−6.521	−2.325	−2.212	0.527	−3.261	−1.162
*F*	b	0.711	1.275	0.558	0.579	−1.828	3.251	0.356	0.637	−0.914	1.626
	c	1.505	1.893	0.795	0.429	−2.265	5.276	0.753	0.946	−1.133	2.638
*Q*	b *	−7.179	2.035	−3.528	0.001	−11.232	−3.125	−3.589	1.017	−5.616	−1.563
	c *	−3.847	1.922	−2.002	0.049	−7.676	−0.019	−1.924	0.961	−3.838	−0.009
*dL*	b	−2.611	2.499	−1.045	0.299	−7.589	2.367	−1.306	1.249	−3.795	1.183
	c	−2.775	2.538	−1.093	0.278	−7.831	2.282	−1.387	1.269	−3.915	1.141
*V* × *F*	b	1.103	0.924	1.194	0.236	−0.737	2.944	0.552	0.462	−0.369	1.472
*V* × *Q*		2.709	1.466	1.848	0.069	−0.211	5.629	1.354	0.733	−0.105	2.814
(*V* × *dL*) *		10.324	2.183	4.730	0.000	5.976	14.672	5.162	1.091	2.988	7.336
(*F* × *Q*) *		5.018	2.081	2.412	0.018	0.873	9.163	2.509	1.040	0.436	4.582
*F* × *dL*		−0.226	1.930	−0.117	0.907	−4.070	3.618	−0.113	0.965	−2.035	1.809
(*Q* × *dL*) *		−7.610	3.268	−2.329	0.023	−14.120	−1.100	−3.805	1.634	−7.060	−0.550

* Statistically significant coefficients.

## Data Availability

The original contributions presented in this study are included in the article. Further inquiries can be directed to the corresponding author.
